# Progesterone Administration Modulates Cortical TLR4/NF-*κ*B Signaling Pathway after Subarachnoid Hemorrhage in Male Rats

**DOI:** 10.1155/2011/848309

**Published:** 2011-03-03

**Authors:** Zhong Wang, Gang Zuo, Xiao-Yong Shi, Jian Zhang, Qi Fang, Gang Chen

**Affiliations:** ^1^Department of Neurosurgery, The First Affiliated Hospital of Soochow University, Suzhou 215006, China; ^2^Department of Neurology, The First Affiliated Hospital of Soochow University, Suzhou 215006, China

## Abstract

Our previous study concerning brain trauma has shown that progesterone could regulate toll-like receptor 4 (TLR4) and nuclear factor-kappa B (NF-*κ*B) signaling pathway in the brain, which also has been proved to play important roles in early brain injury (EBI) after subarachnoid hemorrhage (SAH). The aim of the current study was to investigate whether progesterone administration modulated TLR4/NF-*κ*B pathway signaling pathway in the brain at the early stage of SAH. All SAH animals were subjected to injection of 0.3 ml fresh arterial, non-heparinized blood into prechiasmatic cistern in 20 seconds. Male rats were given 0 or 16 mg/kg injections of progesterone at post-SAH hours 1, 6, and 24. Brain samples were extracted at 48 h after SAH. As a result, SAH could induce a strong up-regulation of TLR4, NF-*κ*B, pro-inflammatory cytokines, MCP-1, and ICAM-1 in the cortex. Administration of progesterone following SAH could down-regulate the cortical levels of these agents related to TLR4/NF-*κ*B signaling pathway. Post-SAH progesterone treatment significantly ameliorated the EBI, such as the clinical behavior scale, brain edema, and blood-brain barrier (BBB) impairment. It was concluded that post-SAH progesterone administration may attenuate TLR4/NF-*κ*B signaling pathway in the rat brain following SAH.

## 1. Introduction

Early brain injury (EBI) is the most common cause of disability and death in patients suffering from aneurysmal subarachnoid hemorrhage (SAH) [1]. Treatment of EBI is considered as a major goal in the management of patients surviving SAH. However, the exact molecular mechanism of EBI still remains obscure, which has hindered the development of effective and specific treatment paradigms for EBI. The term EBI refers to the immediate injury to the brain as a whole, within the first 48 h of the ictus, secondary to SAH [2]. Previous reports [3, 4] have demonstrated that inflammatory responses mediated mainly by the activation of toll-like receptor 4 and nuclear factor-kappa B (TLR4/NF-*κ*B) signaling pathway played an important role in the pathogenesis of EBI following SAH. In their study, the authors mentioned that E5564 (TLR4 inhibitor) could correct the neurological deficits and down-regulate inflammation by inhibiting activation of the TLR4/NF-*κ*B signaling in prechiasmatic blood injection model.

There are now 10 mammalian toll-like receptors (TRLs) identified by sequence analysis. Among them, TRL4, which was widely expressed in brain, can detect endogenous agonists, such as the degradation products of macromolecules, heat shock protein 60 and 70, products of proteolytic cascades, intracellular components of ruptured cells, and products of genes that are activated by inflammation [5]. Furthermore, TLR4 has been demonstrated to play an important role in initiating the cerebral inflammation related to stroke, Alzheimer's disease, Huntington's disease, and Parkinson's disease [6]. All TLRs activate a common signaling pathway that culminates in the activation of NF-*κ*B transcription factors and the mitogen-activated protein kinases (MAPKs). As one of the most important downstream molecules in TLR4 signaling pathway, NF-*κ*B is a transcriptional factor required for the gene expression of many inflammatory mediators, such as interleukin-1*β* (IL-1*β*), tumor necrosis factor-*α* (TNF-*α*), interleukin-6 (IL-6), intercellular adhesion molecule-1 (ICAM-1), and monocyte chemoattractant protein-1 (MCP-1).

Recently, several reports from clinical and experimental studies have demonstrated that progesterone plays neuroprotective roles in traumatic brain injury (TBI) and ischemic brain injury [7] including reducing cerebral edema, preventing neuronal loss, and improving functional outcomes [8]. At the same time, our previous studies [9, 10] have proved that post-TBI progesterone administration attenuated the TLR4/NF-*κ*B signaling pathway in injured rat brain, which was a mechanism whereby progesterone improved the outcome following TBI. Nevertheless till now, it is still unknown whether progesterone can influence TLR4/NF-*κ*B pathway in the brain after SAH. Thus, the aim of the current study was to determine whether progesterone could attenuate the SAH induced activation of TLR4/NF-*κ*B signaling pathway in the cortex. We hypothesized that the effect of progesterone on modulating TLR4/NF-*κ*B signaling pathway could be a mechanism by which progesterone promoted behavioral recovery after SAH.

## 2. Materials and Methods

### 2.1. Animals

The animal use and care protocols, including all operation procedures, were approved by the Animal Care and Use Committee of Soochow University and conformed to the Guide for the Care and Use of Laboratory Animals by the National Institute of Health. Ninety-six male Sprague Dawley rats weighing from 280 to 350 g were purchased from the Animal Center of the Chinese Academy of Sciences (Shanghai, China). They were acclimated in a humidified room and maintained on the standard pellet diet at the Animal Center of Soochow University for 10 days before the experiment. The temperature in both the feeding room and the operation room was maintained at about 25°C.

### 2.2. Prechiasmatic Cistern SAH Model

**Figure 1:**
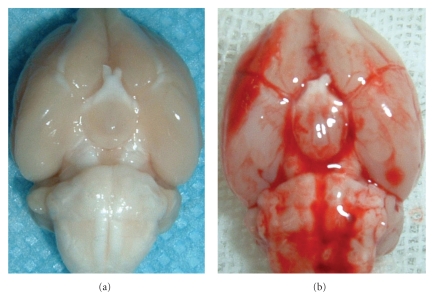
Schematic representation of the areas taken for assay. (a) Control rat brain; (b) SAH rat brain.

Following intraperitoneal anesthesia with 4% chloral hydrate (400 mg/kg body weight), animal head was fixed in the stereotactic frame. Experimental SAH model was produced using stereotaxic insertion of a needle with a rounded tip and a side hole into the prechiasmatic cistern according to our previous study [11]. The needle was tilted 45° in the sagittal plane, and placed 7.5 mm anterior to bregma in the midline, with the hole facing the right side. It was lowered until the tip reached the base of the skull, 2-3 mm anterior to the chiasma (about 10 to 12 mm from the brain surface) and retracted 0.5 mm. Loss of CSF and bleeding from the midline vessels were prevented by plugging the burr hole with bone wax before inserting the needle. The amount of 0.3 mL nonheparinized fresh autologous arterial blood was slowly injected into the prechiasmatic cistern for 20 seconds under aseptic technique. Control animals were injected with 0.3 mL saline. The animals were allowed to recover 45 min after SAH. After operation procedures, the rats were then returned to their cages and the room temperature kept at 23 ± 1°C. 20 mL of 0.9% NaCl was injected subcutaneously right after the operation to prevent dehydration. Heart rate and rectal temperature were monitored, and the rectal temperature was kept at 37 ± 0.5°C, by using physical cooling (ice bag) when required, throughout experiments. It was observed in the present study that inferior basal temporal lobe was always stained by blood. Hence, the brain tissue adjacent to the clotted blood was taken to analysis in our study (Figure 1). Control animals underwent exactly the same procedure as described above with the exception that no blood was injected intracisternally.

### 2.3. Experimental Design

The experimental groups consisted of control (*n* = 6), SAH (*n* = 6), SAH + vehicle (*n* = 6), and SAH + progesterone (*n* = 6). Rats of SAH + progesterone group received injections of 16 mg/kg progesterone (4-Pregnene-3, 20-dione, Sigma-Aldrich Inc., St. Louis, MO, USA) at post-SAH hours 1, 6, and 24 (intraperitoneally for the first and subcutaneously for the remaining two). Rats of SAH + vehicle group received equal volumes of vehicle (2-hydroxypropyl-*β*-cyclodextrin, Sigma-aldrich Inc., St. Louis, MO, USA) [9]. The animals were decapitated at 48 h after SAH for tissue assays. The brain tissue was dissected on ice as described in our previous study [12], some of which was put into 10% buffered formalin, the others were stored at liquid nitrogen immediately until use.

### 2.4. Western Blot Analysis

The frozen brain samples were mechanically lysed in 20 mM Tris, pH 7.6, which contains 0.2% SDS, 1% Triton X-100, 1% deoxycholate, 1 mM phenylmethylsulphonyl fluoride (PMSF), and 0.11 IU/mL aprotinin (all purchased from Sigma-Aldrich, Inc., St. Luis, MO, USA). Lysates were centrifuged at 12,000×g for 20 min at 4°C. The protein concentration was estimated by the Bradford method using the Nanjing Jiancheng (NJJC) protein assay kit (Nanjing Jiancheng Bioengineering Institute, Nanjing, China). The samples (60 *μ*g per lane) were separated by 8% SDS-PAGE and electrotransferred onto a polyvinylidene-difluoride (PVDF) membrane (Bio-Rad Lab, Hercules, CA, USA). The membrane was blocked with 5% skimmed milk for 2 h at room temperature, incubated overnight at 4°C with primary antibodies directed against TLR4, phosphorylation-NF-*κ*B, ICAM-1, and MCP-1 (all from Santa Cruz Biotechnology, Santa Cruz, CA, USA) at the dilutions of 1 : 200, 1 : 150, 1 : 200, and 1 : 150, respectively. The glyceraldehyde-3-phosphate dehydrogenase (GAPDH) (diluted in 1 : 6000, Sigma-Aldrich, Inc., St. Luis, MO, USA) was used as a loading control. After the membrane was washed for 10 min each for six times in PBS+Tween 20 (PBST), it was incubated in the appropriate HRP-conjugated secondary antibody (diluted 1 : 400 in PBST) for 2 h. The blotted protein bands were visualized by enhanced chemiluminescence (ECL) Western blot detection reagents (Amersham, Arlington Heights, IL, USA) and were exposed to X-ray film. Developed films were digitized using an Epson Perfection 2480 scanner (Seiko Corp, Nagano, Japan). Optical densities were obtained using Glyko Bandscan software (Glyko, Novato,CA, USA). The tissue of six animals was used for Western blot analysis at each time point. All experiments have been repeated at least three times.

### 2.5. Immunohistochemical Study

Immunohistochemistry on formalin-fixed paraffin-embedded sections was performed to determine the immunoreactivity of TLR4, NF-*κ*B, and MCP-1. Sections were deparaffinized and rehydrated in graded concentrations of ethanol to distilled water. Endogenous peroxidase activity was blocked with 3% H_2_O_2_ for 5 min, followed by a brief rinse in distilled water and a 15 min wash in PBS. Sections were placed in 10 mmol/L citrate buffer (pH 6.0), and heated in microwave oven at 95°C for 30 min. Sections were cooled at room temperature for 20 min and rinsed in PBS. Nonspecific protein binding was blocked by 40 min incubation in 5% horse serum. Sections were incubated with primary antibodies (anti-TLR4, anti-phosphorylation-NF-*κ*B, and anti-MCP-1, all diluted 1 : 200, from Santa Cruz Biotechnology, Inc., California, USA) for 1 h at room temperature, followed by a 15 min wash in PBS. Sections were incubated with horseradish peroxidase (HRP)-conjugated IgG (1 : 500 dilution, Santa Cruz Biotechnology, Inc., California, USA) for 60 min at room temperature. DAB was used as chromogen and counterstaining was done with hematoxylin. Sections incubated in the absence of primary antibody were used as negative controls.

### 2.6. Enzyme-Linked Immunosorbent Assay (ELISA)

The frozen brain tissue was homogenized with a glass homogenizer in 1 mL of buffer containing 1 mmol/L of PMSF, 1 mg/L of pepstatin A, 1 mg/L of aprotinin, and 1 mg/L of leupeptin in PBS solution (pH 7.2) and centrifuged at 12,000 g for 20 min at 4°C. The cerebral levels of inflammatory mediators were quantified using specific ELISA kits for rats according to the manufacturers' instructions (TNF-*α* from Diaclone Research, France; IL-1*β*, IL-6 from Biosource Europe SA, Belgium) [13]. The cytokine contents in the cortex tissue were expressed as nanograms of cytokines per gram of protein.

### 2.7. Neurologic Scoring

Three behavioral activity examinations (Table 1) were performed at 24 hours after SAH using the scoring system reported previously to record appetite, activity and neurological deficits [14].

### 2.8. Brain Water Content

Brain edema was determined using the wet/dry method as previously described where % brain water = [(wet weight − dry weight)/wet weight] × 100%. Briefly, brain samples were rapidly removed from the skull and placed separately into preweighed and labeled glass vials and weighed. The vials were then placed in an oven for 72 h at 100°C and then reweighed to obtain dry weight content. The number of animals used in each group for brain edema study was control (*n* = 6), SAH (*n* = 6), SAH + vehicle (*n* = 6) and SAH + progesterone (*n* = 6).

### 2.9. Blood-Brain Barrier Permeability

Blood-brain barrier (BBB) permeability was determined by Evans blue (EB) extravasation at 48 h after SAH. Briefly, 2% Evans blue was injected intravenously at a dose of 2 mL/kg. Animals were then reanesthetized after 1 h with urethane (1000 mg/kg) and perfused using saline to remove intravascular EB dye. Animals were then decapitated, the brains removed and homogenized in phosphate buffered saline. Trichloroacetic acid was then added to precipitate protein, and the samples were cooled and centrifuged. The resulting supernatant was measured for absorbance of EB at 610 nm using a spectrophotometer. The number of animals used in each group for brain edema study was control (*n* = 6), SAH (*n* = 6), SAH + vehicle (*n* = 6) and SAH + progesterone (*n* = 6).

### 2.10. Statistical Analysis

All data except neurologic scoring were presented as mean ± SD. SPSS 12.0 was used for statistical analysis of the data. The Mann-Whitney *U*-test was used to compare the behavior and activity score among groups. The other measurements were analyzed by one-way analysis of variance (ANOVA) or Student's *t*-test. Statistical significance was inferred at *P* < .05.

## 3. Results

### 3.1. General Observation

There were no significant differences in body weight, temperature, or injected arterial blood gas data among the experimental groups (*P* > .05, ANOVA, data not shown). After induction of SAH, all animals will stop breathing for about 30 seconds. The mortality rate of rats in the control group was 0 (0/18 rats), and it was 31% (24/78 rats) in the SAH group.

### 3.2. Western Blot Analysis for Detecting TLR4, NF-*κ*B, ICAM-1, and MCP-1 Expressions after SAH

**Figure 2:**
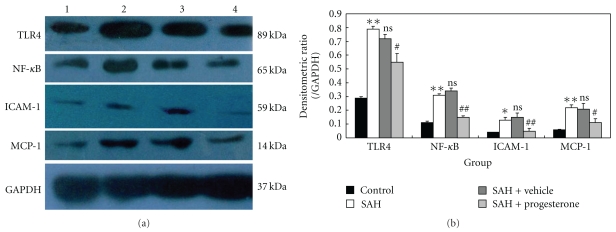
(a) Representative autoradiogram of TLR4, NF-*κ*B, ICAM-1, and MCP-1 in the cortex. We observed TLR4 at 89 kDa, NF-*κ*B at 65 kDa, ICAM-1 at 59 kDa, MCP-1 at 14 kDa, and a loading control GAPDH at 37 kDa. Lane 1, control group; Lane 2, SAH groups; Lane 3, SAH + vehicle group; Lane 4, SAH + progesterone group. It shows that the expression of TLR4/NF-*κ*B related proteins increased after SAH and inhibited by progesterone administration. (b) Quantitative analysis of the Western blot results for TLR4, NF-*κ*B, ICAM-1, and MCP-1. It shows that the levels of TLR4, NF-*κ*B, ICAM-1, and MCP-1 in SAH groups are significantly higher than that in control. After progesterone administration, the TLR4/NF-*κ*B related agents were significantly down-regulated as compared with SAH + vehicle group. Bars represent the mean ± SD (*n* = 6, each group). *P < .05 compared with control group, **P < .01 compared with control group, ^#^ *P* < .05 compared with SAH + vehicle group, ^##^ *P* < .01 compared with SAH + vehicle group, ^ns^ *P* > .05 compared with SAH group.

The protein levels of TLR4, NF-*κ*B, ICAM-1, and MCP-1 were detected by western blot. These proteins were expressed at a low level in the rat brains of control group. The levels of TLR4, NF-*κ*B, ICAM-1, and MCP-1 were significantly increased in the cortex in SAH group as compared with that of sham-operated groups (*P* < .01). The protein expressions had no significant difference between SAH group and SAH + vehicle group (*P* > .05). The expressions of TLR4, NF-*κ*B, ICAM-1, and MCP-1 in the brains of SAH + progesterone group were significantly lower than those of the SAH + vehicle group (Figure 2).

### 3.3. Immunohistochemistry for TLR4, NF-*κ*B, and MCP-1 Expressions after SAH

**Figure 3:**
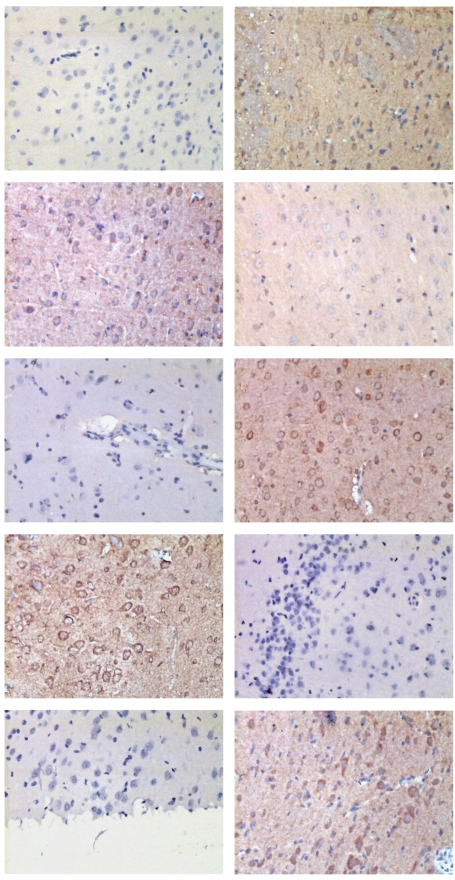

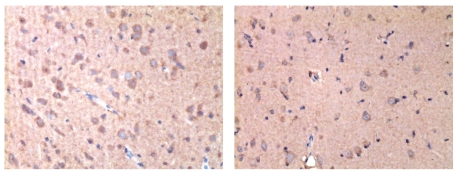
Immunohistochemical study of TLR4, NF-*κ*B, and MCP-1 on brain samples in (scale bar, 100 *μ*m). The arrows show the positive neurons.

To assess the localization of TLR4/NF-*κ*B pathway, immunohistochemistry for TLR4, NF-*κ*B, and MCP-1 was performed. A few TLR4, NF-*κ*B, or MCP-1 positive cells were observed in the control group, which indicates the constitutional activity of TLR4/NF-*κ*B pathway in the control brain of rats (Figure 3). Increased positive cells in the SAH groups could be found in the brain samples (Figure 3). These proteins' immunoreactivity was mainly present in neurons and a little in glia cells (Figure 3). In SAH + progesterone group, the number of positive cells was decreased (Figure 3).

### 3.4. Effect of Progesterone on Concentrations of IL-*β*, TNF-*α* and IL-6 in SAH Brains

**Figure 4:**
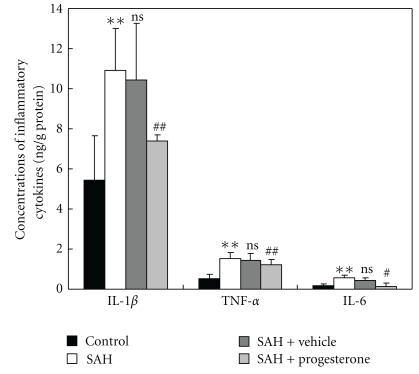
Changes of inflammatory mediators in the injured brains as determined by ELISA in control group (*n* = 6), SAH group (*n* = 6), SAH + vehicle group (*n* = 6), and SAH + progesterone group (*n* = 6). SAH could induce the significantly increased concentrations of IL-1*β*, TNF-*α* and IL-6 in the rat brain surrounding the blood clot. In SAH + progesterone group, the cortical concentrations of IL-1*β*, TNF-*α* and IL-6 were markedly down-regulated as compared with that of SAH + vehicle group. Bars represent the mean ± SD (*n* = 6, each group). **P < .01 compared with control group, ^#^ *P* < .05 compared with SAH + vehicle group, ^##^ *P* < .01 compared with SAH + vehicle group, ^ns^ *P* > .05 compared with SAH group.

Concentrations of IL-1*β*, TNF-*α* and IL-6 were low in the rat brains of control group (5.39 ± 2.18, 0.49 ± 0.19 and 0.18 ± 0.06 ng/g protein, resp.,) (Figure 4). Compared with control group, cortical levels of the three inflammatory cytokines were greatly induced after SAH. As shown in Figure 4, progesterone administration after SAH could lead to significantly decreased IL-1*β*, TNF-*α* and IL-6 concentrations.

### 3.5. Impact of Progesterone on Clinical Behavior Function after Experimental SAH

As compared with control group, clinical behavior function impairment caused by SAH was evident in SAH subjects (*P* < .01, Table 2). No significant difference was seen between the SAH group and SAH + vehicle group (*P* > .05). Progesterone treated rats showed better performance in this scale system than vehicle-treated rats at 24 h after SAH (Table 2), and the difference was statistically significant (*P* < .01, Table 2).

### 3.6. Progesterone-Ameliorated Cerebral Oedema after Experimental SAH

**Figure 5:**
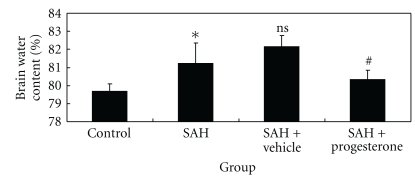
Alterations in brain water content in control group (*n* = 6), SAH group (*n* = 6), SAH + vehicle group (*n* = 6), and SAH + progesterone group (*n* = 6). The brain water content was increased significantly at 48 h after SAH. The progesterone treatment markedly reduced brain water content. No difference of brain water content was detected between SAH and SAH + vehicle groups *P < .05 versus control group, ^#^ *P* < .05 versus SAH + vehicle group, ^ns^ *P* > .05 versus SAH group.

Significant increase (*P* < .05) in water content was detected in the brain samples at 48 h after SAH when compared with rats in control group (Figure 5). The mean value of brain water content in the cortex was decreased by progesterone administration (*P* < .05) as compared with SAH + vehicle group. The results suggested that progesterone treatment could attenuate brain edema in this rat SAH model.

### 3.7. Influence of Progesterone on the Blood-Brain Barrier Permeability Following SAH

**Figure 6:**
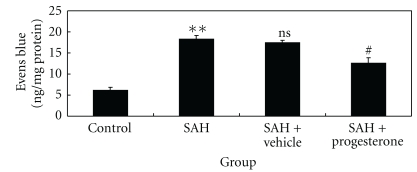
Alterations in Evans blue extravasation in control group (*n* = 6), SAH group (*n* = 6), SAH + vehicle group (*n* = 6), and SAH + progesterone group (*n* = 6). SAH could induce a marked increase of BBB extravasation in the rat brain compared with control group. After progesterone administration, the Evans blue extravasation was significantly reduced as compared with SAH + vehicle group. **P < .01 versus control group, ^#^ *P* < .05 versus SAH + vehicle group, ^ns^ *P* > .05 versus SAH group.

The pattern of Evans blue extravasation following SAH is shown in Figure 6. Rats in SAH + vehicle group demonstrated a significant increase (*P* < .01) in BBB permeability to Evans blue relative to rats of control group. Administration of progesterone significantly inhibited Evans blue extravasation (*P* < .05), indicating a reduced BBB opening in response to progesterone treatment.

## 4. Discussion

The main findings of this study are as follows: (1) TLR4 and NF-*κ*B proteins were up regulated remarkably following SAH; (2) the levels of IL-*β*, TNF-*α*, IL-6, ICAM-1 and MCP-1 in the brain were significantly increased after prechiasmatic blood injection; (3) the cortical levels of these agents related to TLR4/NF-*κ*B signaling pathway were suppressed when treated with progesterone; (4) after progesterone administration, EBI was significantly ameliorated. These findings suggest for the first time that progesterone may attenuate the SAH-induced TLR4/NF-*κ*B signaling pathway activation that may facilitate the development of EBI following SAH in rats.

The researchers began to examine the direct role of progesterone as a therapeutic agent in brain injury by trying to account for observations that females sometimes recover better from TBI compared with males and it was proved by an experimental animal study from Attella et al. [15] and a clinical study from Groswasser et al. [16]. Over the past 20 years, several animal studies about TBI have suggested that progesterone modulated excitoxicity [17–19], reconstituted the blood brain barrier [20, 21], reduced cerebral edema [22, 23], decreased neuronal loss, and enhanced recovery [24]. Moreover, Clinical trials [25, 26] also showed that moderate brain trauma survivors who received progesterone were more likely to have a moderate to good outcome than those randomized to placebo and no serious adverse events were attributed to progesterone. In this current study, we found that progesterone administration following SAH could improve clinical behavior function and decrease the degree of brain edema and BBB impairment, which was never demonstrated in the previous research. And also, none of previous studies focused on the influence of progesterone on TLR4/NF-*κ*B signaling pathway related to cerebral inflammation after SAH.

The family of TLRs plays a key role in controlling innate immunity that responds to a wide variety of pathogen-associated molecules [27]. A variety of TLRs have been identified in human cells and some other species' brain [28, 29]. Several studies have suggested that TLR4 is critical for lipopolysaccharide-induced injury in the central nervous system [28, 30]. Both endothelial and smooth muscle cells of the blood vessels in the brain also express TLR4 and can respond to stimulation [31, 32]. Thus the TLR4 is well positioned in the central nervous system and seemed possibly to initiate inflammation following SAH. The TLRs-mediated intracellular signaling pathways converge to activate NF-*κ*B and c-Jun N-terminal kinases (JNKs), which induce the transcription of a series of cytokine/chemokine genes that are involved in the initiation or regulation of the inflammatory response. Although the results of the present study suggested that the level of TLR4/NF-*κ*B pathway in the brain was increased following SAH and could be suppressed after progesterone administration, the potential mechanism underlying the initial effect on TLR4/NF-*κ*B signaling pathway following SAH remains unclear.

As mentioned in Section 1, a lot of endogenous ligands have been found for TLRs, including heat shock proteins, double-strand RNA, high-mobility group box 1 (HMGB1) protein, surfactant proteins A and D, hyaluronan and fibrinogen [33], some of which have been reported to become present or elevated in the CSF and brain after SAH [34, 35]. However, the principal TLR4 ligand in the brain after SAH and the mechanisms responsible for the beneficial effects of progesterone call for further research. These TLRs ligands were released from damaged, dead or ruptured cells in response to tissue damage, injury and/or infection [35]. The TLR4/NF-*κ*B pathway activation is thought, at least in part, to be caused by the extravasated blood, the acute CBF reduction and the induced increase in necrotic cells. We tentatively put forward that the endogenous danger signals released from extravasated blood in EBI might be recognized through TLR4 and perhaps other TLRs to initiate inflammatory responses. 

To the best of our knowledge, this is the first study to demonstrate the effect of progesterone on the TLR4/NF-*κ*B signaling pathway in the brain after SAH. We found that SAH could upregulate expressions of TLR4 and NF-*κ*B, levels of IL-*β*, TNF-*α* and IL-6, MCP-1, and ICAM-1 expression in the surrounding brain of blood clot, which could be markedly inhibited by progesterone administration. These results suggest that SAH could induce an activation of TLR4/NF-*κ*B signaling pathway that might play a central role in the inflammatory response that leads to secondary insults after SAH. The therapeutic benefit of post-SAH progesterone administration might be due to its salutary effect on modulating TLR4/NF-*κ*B signaling pathway.

## Figures and Tables

**Table 1:** Behavior and activity scores.

Category	Behavior	Score
Appetite	Finished meal	0
	Left meal unfinished	1
	Scarcely ate	2

Activity	Walk and reach at least three corners of the cage	0
	Walk with some stimulations	1
	Almost always lying down	2

Deficits	No deficits	0
	Unstable walk	1
	Impossible to walk	2

**Table 2:** Clinical behavior scales in each group.

Group	Mean Score
Control (*n* = 6)	0.5
SAH (*n* = 6)	2.5**
SAH + vehicle (*n* = 6)	2.7^ns^
SAH + progesterone (*n* = 6)	1.7^##^

**P < .01 compared with control group.

^ns^ *P* > .05 compared with SAH group.

^##^ *P* < .01 compared with SAH + vehicle group.
