# Correction to “Progesterone Administration Modulates Cortical TLR4/NF‐κB Signaling Pathway after Subarachnoid Hemorrhage in Male Rats”

**DOI:** 10.1155/mi/9798459

**Published:** 2026-03-06

Z. Wang, G. Zuo, X.‐Y. Shi, J. Zhang, Q. Fang, and G. Chen, “Progesterone Administration Modulates Cortical TLR4/NF‐κB Signaling Pathway after Subarachnoid Hemorrhage in Male Rats,” *Mediators of Inflammation*, 2011, 848309, https://doi.org/10.1155/2011/848309.

The original article did not include a reference to a previous work, which used the same schematic diagram shown in Figure 1. The authors would like to add a citation to their earlier work [1] in the legend of Figure 1. The corrected Figure 1 legend is shown below:

Schematic representation of the areas taken for assay. (a) Control rat brain; (b) SAH rat brain [1].

We apologize for this error.
